# Identification of a Polyketide Synthase Gene Responsible for Ascochitine Biosynthesis in *Ascochyta fabae* and Its Abrogation in Sister Taxa

**DOI:** 10.1128/mSphere.00622-19

**Published:** 2019-09-25

**Authors:** Wonyong Kim, Judith Lichtenzveig, Robert A. Syme, Angela H. Williams, Tobin L. Peever, Weidong Chen

**Affiliations:** a Department of Plant Pathology, Washington State University, Pullman, Washington, USA; b Korean Lichen Research Institute, Sunchon National University, Suncheon, Jeonnam, South Korea; c Centre for Crop and Disease Management, Curtin University, Bentley, Western Australia, Australia; d USDA-ARS Grain Legume Genetics and Physiology Research Unit, Pullman, Washington, USA; Carnegie Mellon University

**Keywords:** *Ascochyta fabae*, Didymellaceae, ascochitine, citrinin, nonsense mutation, polyketide, secondary metabolite

## Abstract

The polyketide-derived secondary metabolite ascochitine is produced by species in the Didymellaceae family, including but not restricted to *Ascochyta* species pathogens of cool-season food legumes. Ascochitine is structurally similar to the well-known mycotoxin citrinin and exhibits broad-spectrum phytotoxicity and antimicrobial activities. Here, we identified a polyketide synthase (PKS) gene (denoted *pksAC*) responsible for ascochitine production in the filamentous fungus Ascochyta fabae. Deletion of the *pksAC* prevented production of ascochitine and its derivative ascochital in A. fabae. The putative ascochitine biosynthesis gene cluster comprises 11 genes that have undergone rearrangement and gain-and-loss events relative to the citrinin biosynthesis gene cluster in Monascus ruber. Interestingly, we also identified *pksAC* homologs in two recently diverged species, A. lentis and A. lentis var. *lathyri*, that are sister taxa closely related to ascochitine producers such as A. fabae and A. viciae-villosae. However, nonsense mutations have been independently introduced in coding sequences of the *pksAC* homologs of A. lentis and A. lentis var. *lathyri* that resulted in loss of ascochitine production. Despite its reported phytotoxicity, ascochitine was not a pathogenicity factor in A. fabae infection and colonization of faba bean (Vicia faba L.). Ascochitine was mainly produced from mature hyphae at the site of pycnidial formation, suggesting a possible protective role of the compound against other microbial competitors in nature. This report highlights the evolution of gene clusters harnessing the structural diversity of polyketides and a mechanism with the potential to alter secondary metabolite profiles via single nucleotide polymorphisms in closely related fungal species.

**IMPORTANCE** Fungi produce a diverse array of secondary metabolites, many of which are of pharmacological importance whereas many others are noted for mycotoxins, such as aflatoxin and citrinin, that can threaten human and animal health. The polyketide-derived compound ascochitine, which is structurally similar to citrinin mycotoxin, has been considered to be important for pathogenicity of legume-associated *Ascochyta* species. Here, we identified the ascochitine polyketide synthase (PKS) gene in Ascochyta fabae and its neighboring genes that may be involved in ascochitine biosynthesis. Interestingly, the ascochitine PKS genes in other legume-associated *Ascochyta* species have been mutated, encoding truncated PKSs. This indicated that point mutations may have contributed to genetic diversity for secondary metabolite production in these fungi. We also demonstrated that ascochitine is not a pathogenicity factor in A. fabae. The antifungal activities and production of ascochitine during sporulation suggested that it may play a role in competition with other saprobic fungi in nature.

## Abstract (precis)

Fungi produce a diverse array of secondary metabolites, many of which are of pharmacological importance whereas many others are noted for mycotoxins, such as aflatoxin and citrinin, that can threaten human and animal health. The polyketide-derived compound ascochitine, which is structurally similar to citrinin mycotoxin, has been considered to be important for pathogenicity of legume-associated *Ascochyta* species. Here, we identified the ascochitine polyketide synthase (PKS) gene in Ascochyta fabae and its neighboring genes that may be involved in ascochitine biosynthesis. Interestingly, the ascochitine PKS genes in other legume-associated *Ascochyta* species have been mutated, encoding truncated PKSs. This indicated that point mutations may have contributed to genetic diversity for secondary metabolite production in these fungi. We also demonstrated that ascochitine is not a pathogenicity factor in A. fabae. The antifungal activities and production of ascochitine during sporulation suggested that it may play a role in competition with other saprobic fungi in nature.

## INTRODUCTION

Polyketides are the most abundant fungal secondary metabolites and have been studied for centuries for either their beneficial bioactivity or their toxicity. The genes underlying the biosynthetic pathways for these and other secondary metabolites are arranged in contiguous clusters on the genome (1). Linking of the biosynthetic pathways to their respective gene clusters has been facilitated by technological advances in metabolomics and genomics.

Ascochitine, also known as ascochytine, is an *o*-quinone methide produced via a polyketide biosynthetic pathway. Ascochitine was originally reported as a selective antifungal agent, as some tested fungi were more sensitive to ascochitine whereas others were able to detoxify ascochitine (2, 3). In addition, applied externally, ascochitine can cause electrolyte leakage from the leaf disc of *Clematis* cultivars susceptible to an ascochitine producer, Phoma clematidina. However, *Clematis* cultivars resistant to P. clematidina were largely insensitive to ascochitine, suggesting that ascochitine is a host-selective phytotoxin (4).

Ascochitine was first discovered in culture extracts of Ascochyta pisi Lib. (5), and later in A. fabae Speg. (6), causal agent of Ascochyta blight of pea (Pisum sativum L.) and faba bean (Vicia faba L.), respectively (7). The ability to produce ascochitine is prevalent among legume-associated *Ascochyta* but has been lost in some of the lineages (8). Ascochitine production has been also reported in species in other genera in the family Didymellaceae (4, 9) and in the sister family Pleosporaceae (10) in the Dothideomycetes (11–14). However, ascochitine has not been found in A. lentis, which is placed as a sister taxon to the ascochitine producers such as A. fabae, A. pisi, and A. viciae-villosae (8). Instead, new phytotoxic compounds have been discovered in culture extracts from A. lentis and A. lentis var. *lathyri* (15, 16).

Ascochitine is structurally similar to the well-known mycotoxin citrinin, which is produced by diverse fungal species in the genera *Aspergillus*, *Monascus*, and *Penicillium* in the Eurotiomycetes. The citrinin biosynthesis gene cluster, including a polyketide synthase (PKS) gene that initiates the biosynthetic pathway, as well as a pathway-specific transcription regulator, has been identified previously in *Monascus* spp. (17–21). Ascochitine biosynthetic pathways of ascochitine have been proposed previously (9, 22). However, identification of the underlying gene cluster is lacking.

All described fungal PKSs belong to the type I iterative PKS group that contains several functional domains, and all synthesize structurally diverse compounds, such as the health-threatening aflatoxin, the cholesterol-lowering drug lovastatin, and melanin pigments (23). The type I iterative PKSs can be broadly classified into nonreducing PKS (NR-PKS) and highly reducing PKS (HR-PKS). Many NR-PKSs have an N-terminal starter unit–ACP transacylase (SAT) domain–which mediates the loading of a starter unit, followed by chain extension components ketosynthase (KS), acyl transferase (AT), and acyl carrier protein (ACP). The product template (PT) domain is often located between the AT and ACP domains and may be involved in polyketide chain length determination (24). The C-terminal processing domains of NR-PKS are variable and often include some of the following domains: methyltransferase (MeT), thioesterase (TE), Claisen-cyclase (CLC), thiolester reductase domain (R), and additional ACP domains (24). HR-PKSs often possess dehydratase (DH), ketoreductase (KR), and enoyl reductase (ER) and terminate with an ACP domain, producing complex highly reduced compounds. To date, there are no identified HR-PKSs that have the N-terminal SAT and PT domains commonly found in NR-PKSs. Among the diverse PKS domains, the KS domain has been used in classification of PKSs in fungi due to the high amino acid sequence similarity (25–27).

Since the first discovery of ascochitine in 1956 (5), the compound has been found in many different fungal taxa and researchers have reported its phytotoxicity and antifungal activities. However, direct evidence is lacking for the roles of ascochitine in pathogenicity and competition with other microbes, primarily due to lack of information about the genetic control of biosynthesis. Thus, the objectives of this study were (i) to identify genes responsible for ascochitine biosynthesis and the associated gene cluster in A. fabae, (ii) to examine the roles of ascochitine in the fungal biology through targeted gene knockout study, and (iii) to investigate genetic diversity in ascochitine biosynthetic genes among legume-associated *Ascochyta* species.

## RESULTS

### Homology-based search for putative ascochitine PKS gene.

**FIG 1:**
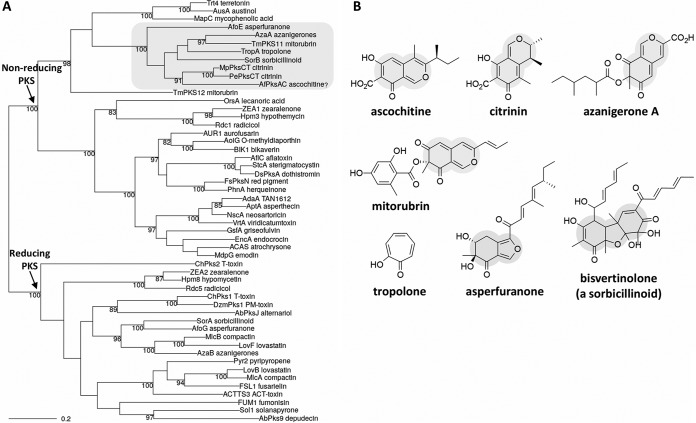
Genealogy of fungal PKS. (A) The maximum likelihood phylogeny was estimated from deduced amino acid sequences of the KS domain of PKS genes with known products. The numbers at the internal nodes indicate percentages of bootstrap support greater than 80% from 1,000 bootstrap replications. Branch lengths are proportional to the inferred amount of evolutionary change, and the scale represents 0.2 amino acid sequence substitutions per site. A subclade that includes *pksCT* and putative ascochitine PKS genes (denoted *pksAC*) was highlighted. (B) Chemical structures of polyketides in the highlighted subclade within the nonreducing PKS clade. The hallmark oxygenated heterocyclic cores are shaded.

To search for a PKS gene responsible for ascochitine biosynthesis, we identified a total of nine putative type I PKS genes from the genome sequence of A. fabae isolate AF1, using the antiSMASH program (28). Then, the KS domain of the citrinin PKS gene (*pksCT*) in Monascus purpureus was queried against the putative PKS genes found in A. fabae. We found a PKS gene (here referred to as *pksAC*) similar to the *pksCT* gene, showing 67% identity in the KS domains at the amino acid sequence level. The PKS domain architectures of the *pksAC* and *pksCT* genes were identical (SAT-KS-AT-PT-ACP-MeT-R). To predict a PKS function in an evolutionary framework, we reconstructed the genealogy of fungal PKSs, using the deduced amino acid sequences of the KS domains of the *pksAC* gene and 51 PKS genes that have been definitively linked to the biosynthesis of specific compounds (see Text S1 in the supplemental material). It seems reasonable to believe that the resulting polyketides produced by phylogenetic orthologs are likely more similar to each other in their chemical structures. As expected, the *pksAC* gene was placed next to the *pksCT* genes in the phylogenetic tree (Fig. 1A), providing additional evidence of the link between the *pksAC* gene and ascochitine biosynthesis. The polyketide products within the subclade that includes the *pksAC* and *pksCT* genes share a common characteristic, the heavily oxygenated heterocyclic core, which is observed most often in pyranoquinones, such as ascochitine, azanigerones, citrinin, and mitorubrin (Fig. 1B).

10.1128/mSphere.00622-19.1 TEXT S1 The deduced amino acid sequences of the KS domains of fungal PKS genes. Download Text S1, DOCX file, 0.02 MB. Copyright © 2019 Kim et al. 2019 Kim et al. This content is distributed under the terms of the Creative Commons Attribution 4.0 International license.

### Characterization of *pksAC* in A. fabae.

**FIG 2:**
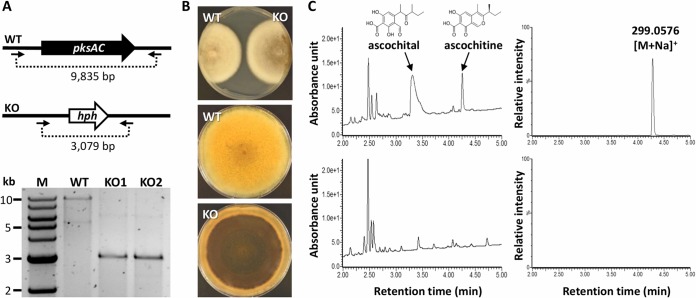
Generation of ascochitine-negative mutants. (A) The *pksAC* coding sequence of A. fabae isolate AF1 (WT) was replaced with a hygromycin phosphotransferase gene cassette (*hph*). Gene replacement was confirmed by PCR; the primer pair is indicated by arrows. (B) A front view of the WT and Δ*pksAC* (KO) strains after 10 days of hyphal growth on potato dextrose agar is shown in the top panel; reverse views of hyphal growth after 20 days are shown in the middle and bottom panels. (C) UV-visible light (UV-Vis) chromatograms (left panels) and selected ion mass chromatograms (right panels) of WT (upper panels) and KO (bottom panels) culture extract. Peaks characteristic of ascochitine and its derivative ascochital are indicated with arrows where present. Denoted is the ascochitine observed *m/z*; for reference, the *m/z* calculated for C_15_H_16_NaO_5_ [M+Na]^+^ is 299.0895.

To validate the involvement of the *pksAC* gene in ascochitine production in A. fabae isolate AF1 (wild type [WT]), we conducted targeted gene replacement of the *pksAC* gene with a hygromycin phosphotransferase gene cassette. Successful replacement in putative transformants was verified with PCR using a primer pair that annealed to flanking regions of the introduced cassette (Fig. 2A). The resulting knockout mutants (Δ*pksAC*) were similar to the WT in growth rate (Fig. 2B; top panel). In the aged culture, the colony of the Δ*pksAC* gene became highly melanized (Fig. 2B), which was likely caused by the excess of acetates that otherwise should have been used in ascochitine biosynthesis. Similar phenomena have been observed in solanapyrone-negative mutants in Ascochyta rabiei (29) and in a mutant defective in a red pigment production in Fusarium neocosmosporiellum (30).

The Δ*pksAC* gene lost the ability to produce ascochitine and its derivatives, as evidenced by the chemical profiles of the culture extracts (Fig. 2C). Liquid chromatography-mass spectrometry (LC-MS) analysis of a culture extract of the WT confirmed the production of ascochitine (eluted at 4.28 min) and ascochital (another end product from the ascochitine biosynthetic pathway; eluted at 3.32 min) (9) (Fig. 2C; top panels). In the Δ*pksAC* gene, there was no detectable production of ascochitine and ascochital, indicating that the *pksAC* gene is required for ascochitine biosynthesis (Fig. 2C; bottom panels).

### Identification of ascochitine biosynthesis gene cluster.

The identity and function of each gene in the citrinin biosynthesis gene cluster in Monascus ruber were recently reported (21). On the basis of the structural similarity between citrinin and ascochitine (Fig. 1B), we hypothesized that the contents of the citrinin and ascochitine gene clusters are conserved. Since the genome assembly of A. fabae isolate AF1 around the *pksAC* locus was not complete, we used the genome sequences of another A. fabae isolate (isolate AF247/15) and an A. viciae-villosae isolate (isolate AV22) that were retrieved from the NCBI database to search for ascochitine biosynthesis gene cluster. There was one nucleotide difference in the *pksAC* sequence in the AF247/15 isolate (G to A at position 5437) that caused a nonsynonymous substitution (valine to isoleucine) relative to the *pksAC* sequences in the AF1 and AF55/01 isolates. By comparing genes neighboring the *pksAC* gene to the gene members of the citrinin gene cluster, we identified a minimal set of conserved genes (*orf1* to *orf11*; Table 1) that are likely involved in ascochitine biosynthesis in the genomes of the AF247/15 and AV22 isolates. The gene content and order of the ascochitine biosynthesis gene cluster in the two ascochitine producers were identical, and genes further downstream of *orf1* or upstream of *orf11* did not have significant sequence similarities with the members of the citrinin gene cluster (not shown).

**TABLE 1:** Annotation of the ascochitine biosynthesis gene cluster in *A. fabae*

Gene	Length (aa)^*a*^	Protein function	Citrinin gene	% aa
*orf1*	494	NAD(P)^+^-dependent aldehyde dehydrogenase	*mrl4*	54
*orf2*	186	Cupin domain-containing protein		
*orf3*	331	Nonheme Fe(II)-dependent oxygenase	*mrl2*	62
*orf4*	270	Serine hydrolase	*mrl1*	53
*pksAC* (*orf5*)	2,645	Nonreducing polyketide synthase (NR-PKS)	*pksCT*	52
*orf6*	517	Transporter	*mrr1*	60
*orf7*	1,886	Highly reducing polyketide synthase (HR-PKS)		
*orf8*	379	SAT domain-containing protein		
*orf9*	621	NAD(P)^+^-dependent oxidoreductase	*mrl7*	60
*orf10*	128	Glyoxylase-like domain-containing protein	*mrl5*	46
*orf11*	573	Transcription factor	*mrl3*	45

a aa, amino acids.

**FIG 3:**
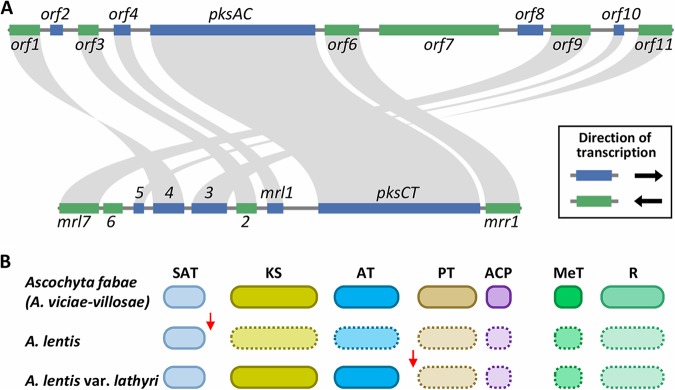
Divergence of ascochitine biosynthesis gene cluster. (A) Synteny plots of ascochitine (top) and citrinin (bottom) biosynthesis gene clusters. Annotations of genes in the citrinin biosynthesis gene cluster are presented as described previously by He and Cox (21). (B) Schematic diagrams of PKS domain structures of *pksAC* orthologs in legume-associated *Ascochyta* species. Arrows indicate lineage-specific SNPs that caused nonsense mutations. Domains surrounded by dashed lines indicate absence of translation due to premature stop codons.

The *orf1*, *orf3*, and *orf9* genes were homologous to the citrinin biosynthesis genes encoding NAD(P)^+^-dependent aldehyde dehydrogenase (*mrl4*), nonheme Fe(II)-dependent oxygenase (*mrl2*), and NAD(P)^+^-dependent oxidoreductase (*mrl7*), respectively. These enzymes were involved in subsequent oxidations of methyl groups to the carboxylic acid of the heterocyclic ring of citrinin (21). The *mrl6* gene, encoding an enzyme involved in the generation of carbonyl groups in the heterocyclic ring of citrinin, was missing in the putative ascochitine gene cluster in both A. fabae and A. viciae-villosae. An additional PKS gene (*orf7*) was found adjacent to the *pksAC* gene (Fig. 3A). This PKS gene is classified as a highly reducing PKS gene. As such, it may be involved in the addition of the *sec*-butyl group in ascochitine via mechanisms analogous to the biosynthesis of asperfuranone and azanigerones in *Aspergillus* spp. (31). The gene cluster also includes a transporter gene (*orf6*) and a gene encoding a putative transcription factor (*orf11*). Overall, despite the observed structural similarity in ascochitine and citrinin, the responsible gene clusters appeared to have diverged through gene order rearrangement and through gene gains and losses (Fig. 3A).

### Sequence diversity in *pksAC* orthologs.

Genetic diversity in secondary metabolite gene clusters can be introduced by different mechanisms in fungi (32). We investigated how ascochitine gene clusters have diverged during the evolution of legume-associated *Ascochyta* species. First, the presence of *pksAC* orthologous genes was checked in A. lentis and A. lentis var. *lathyri*, which are closely related to ascochitine producers such as A. fabae and A. viciae-villosae (33). We surveyed genome sequences of three A. lentis isolates, namely, isolates AL1, AL4, and Kewell, and one A. lentis var. *lathyri* isolate, namely, isolate ER1415, and found orthologous *pksAC* genes largely as complete copies in the four isolates. However, many single nucleotide polymorphisms (SNPs) were observed in the *pksAC* orthologs that included nonsense mutations introducing a premature stop codon that resulted in truncated PKS gene products (Fig. 3B). Interestingly, the nucleotide positions of the first nonsense mutation differed in A. lentis and A. lentis var. *lathyri*: G to T at position 828 in A. lentis and C to T at position 4116 in A. lentis var. *lathyri* (Table 2). The A. lentis genome sequences were all from Australian isolates, so we examined additional isolates of different geographical origins, including Asia, Europe, and South America (Table 2). Since A. lentis var. *lathyri* is an species endemic to Italy, we checked one more isolate collected in a different year (Table 2). The SNPs that caused the nonsense mutation were confirmed in all the tested isolates by Sanger sequencing.

**TABLE 2:** Single nucleotide polymorphisms that caused nonsense mutations in orthologous *pksAC* genes^*a*^

Species (isolate code)	Origin of isolation	Location (collector, yr)	SNP1/ SNP2
Ascochyta fabae (AF1)	Vicia faba	Saskatoon, Canada (A. Vandenberg, 1992) (8, 33)	GGA/CAG
Ascochyta fabae (AF55/01)	Vicia faba	Cochaleechie, Australia (R. Kimber, 2001)	GGA/CAG
Ascochyta fabae (AF247/15)	Vicia faba	Tarlee, Australia (NA)	GGA/CAG
Ascochyta viciae-villosae (AV22)	Vicia villosae	Apriltsi, Bulgaria (W. Kaiser, 1996) (33)	GGA/CAG
Ascochyta lentis (AL1)	Lens culinaris	Australia (W. Kaiser, NA) (8, 33)	**T**GA/CAG
Ascochyta lentis (AL2)	Lens culinaris	Brazil (W. Kaiser, NA) (8, 33)	**T**GA/CAG
Ascochyta lentis (AL3)	Lens culinaris	Canada (W. Kaiser, NA) (8, 33)	**T**GA/CAG
Ascochyta lentis (AL4)	Lens culinaris	Horsham, Australia (M. Nasir and T. W. Bretag, 1998) (64, 65)	**T**GA/CAG
Ascochyta lentis (AL6)	Lens culinaris	Russia (W. Kaiser, NA) (8, 33)	**T**GA/CAG
Ascochyta lentis (AL11)	Lens culinaris	India (W. Kaiser, NA) (8, 33)	**T**GA/CAG
Ascochyta lentis (Kewell)	Lens culinaris	Kewell, Australia (M. Nasir and T. W. Bretag, 2001) (65)	**T**GA/CAG
Ascochyta lentis var. lathyri (ER1415)	Lathyrus sativus	Salerno, Italy (A. Infantino, 2007) (8, 42)	GGA/**T**AG
Ascochyta lentis var. lathyri (ER1478)	Lathyrus sativus	Salerno, Italy (A. Infantino, 2008) (8, 42)	GGA/**T**AG

a SNP1 and SNP2 occurred at nucleotide 828 and 4,116 positions from the A of the ATG translation initiation codon of the *pksAC* gene in A. fabae isolate AF247/15, respectively. NA, not available. Bold letter Ts in column 4 represent nucleotide substitutions to be a stop codon.

To investigate possible selection acting on ascochitine biosynthesis genes, we estimated synonymous and nonsynonymous substitution rates between *pksAC* orthologs. Although the degrees of sequence divergence between *pksAC* orthologs were similar at both the DNA and deduced amino acid sequence levels, the ratio of synonymous to nonsynonymous substitution rates between A. fabae and A. viciae-villosae (ascochitine producers) was slightly higher than between A. fabae and the two ascochitine-negative species (Table 3), which could be ascribed to some degree of purifying selection acting on the *pksAC* genes in the ascochitine producers.

**TABLE 3:** Estimation of synonymous and nonsynonymous substitution rates in *pksAC* orthologs^*a*^

Ascochyta fabae vs.:	No. of Sd	No. of Sn	No. of S	No. of N	Ratio	nt%	aa%
Ascochyta viciae-villosae	107	50	1,787.0	5,875.0	7.04	97.95	98.08
Ascochyta lentis	87	54	1,786.2	5,875.8	5.30	98.16	97.92
Ascochyta lentis var. lathyri	102	57	1,787.2	5,874.8	5.88	97.92	97.77

a Sd, observed synonymous substitutions; Sn, observed nonsynonymous substitutions; S, potential synonymous substitutions; N, potential nonsynonymous substitutions; Ratio, the ratio of synonymous to nonsynonymous substitutions = (Sd/S)/(Sn/N); nt%, percentage of nucleotides; aa%, percentage of amino acids.

### Possible roles of ascochitine.

**FIG 4:**
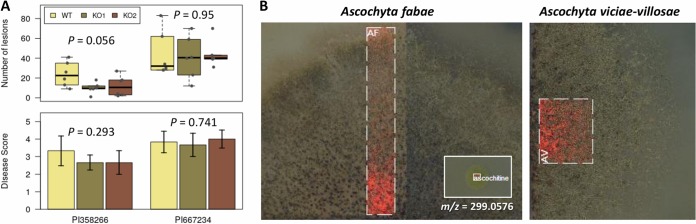
Characterization of ascochitine-negative mutants. (A) Pathogenicity tests were performed on faba bean strains (accession no. PI 358266, moderately resistant; accession no. PI 667234, susceptible). *P* values are for one-way ANOVA for each strain. (B) *In situ* detection of ascochitine in the cultures of A. fabae (AF) and A. viciae-villosae (AV). A selected area (dashed box) was analyzed with a Fourier-transform ion cyclotron resonance mass spectrometry instrument. Detection of ascochitine (*m/z* calculated for C_15_H_16_NaO_5_ [M+Na]^+^, 299.0895; observed, 299.0576). The inset shows an ascochitine standard spotted onto a separate place, and reference mass spectrometry signals were obtained in a simultaneous run. Small black dots visible on the fungal colony represent pycnidia (asexual fruiting bodies). The difference in brightness of the left and right side of the A. fabae colony is due to the background of the embedded microscopic slide on which the fungus grew.

Ascochitine has been postulated to represent a pathogenicity factor (4). Thus, the role of ascochitine in the A. fabae and faba bean pathosystem was evaluated by comparing the WT and the Δ*pksAC* mutant with respect to their disease-causing abilities. Faba bean strains with accession numbers PI 358266 (moderately resistant) and PI 667234 (susceptible) served as hosts. The Δ*pksAC* mutants maintained the ability to cause disease on faba bean, and there was no significant difference between the WT and the Δ*pksAC* mutant with respect to disease incidence or severity (one-way analysis of variance [ANOVA] *P > *0.05; Fig. 4A). These results suggested that ascochitine production is not essential for pathogenicity in A. fabae on faba bean.

matrix-assisted laser desorption ionization (MALDI) imaging mass spectrometry enabled the detection of ascochitine on the surface of the fungal colony, and ascochitine accumulated in aged hyphae where pycnidia (the asexual fruiting bodies) formed (Fig. 4B), suggesting that ascochitine was associated with sporulation and might play a protective role against competing microbes in nature.

## DISCUSSION

With the advent of high-throughput sequencing technologies, fungal genomes are becoming available in exponentially increasing numbers (34). Nevertheless, relatively few secondary metabolite pathways have been linked to their respective biosynthetic gene clusters. Although classes of compounds can be made through completely distinct biochemical pathways, phylogenetic orthologs that encode enzymes with identical or nearly identical biochemical functions are expected to produce similar compounds. Therefore, homology-based prediction within an evolutionary framework can facilitate linking of genes to molecules, and, ultimately, the genetic information can be used for developing heterologous systems for expression of compounds of pharmaceutical and industrial interest in the postgenomic era (31, 35).

To date, ascochitine has been found in many species in legume-associated *Ascochyta* and other genera in the Dothideomycetes, such as *Calophoma*, *Phoma*, *Pleospora*, and *Stagonosporopsis* (4, 8–10). Recent efforts to resolve the structure of complex Didymellaceae, including *Ascochyta* and *Phoma* taxa in the Dothideomycetes, reclassified P. clematidina into *Calophoma*, which is a sister genus to the *Ascochyta*, and A. salicorniae into *Stagonosporopsis* (11, 13, 14). *Ascochyta hyalospora* was also renamed Pleospora chenopodii, which belongs to the Pleosporaceae (12). These widespread distributions of ascochitine production in the Dothideomycetes suggested that the trait of producing ascochitine is ancestral to legume-associated *Ascochyta* species. Despite the phylogenetic distance between ascochitine- and citrinin-producing fungi, the ascochitine and citrinin biosynthetic genes exhibited high degrees of sequence similarities (up to 62%), suggesting a common origin of ascochitine and citrinin gene clusters. The two gene clusters showed gene content polymorphisms and appear to have experienced multiple gene translocation and inversion events during divergence from the gene cluster that shared the most recent common ancestry. Interestingly, the ascochitine gene cluster included a HR-PKS (*orf7*) with an unusual PKS domain architecture (KS-DH-ER-KR-ACP), missing AT and MeT domains found in many HR-PKSs, such as lovastatin diketide synthase *lovF* (KS-AT-DH-MeT-ER-KR-ACP) (36). Most PKS require an AT domain to transfer acyl groups to the KS and ACP components. It is currently unknown whether the *orf7* without an AT domain is truly functional. Since the *orf7* ortholog was also found in A. viciae-villosae, it is less likely that the unusual PKS architecture of the *orf7* was due to genome assembly or annotation errors. Thus, the precise role of the HR-PKS in ascochitine biosynthesis needs to be determined.

It was previously hypothesized that a reduced diketide is used as a starter unit for citrinin biosynthesis by the *pksCT* gene (37). Recently, however, He and Cox demonstrated that acetate is used as a starter unit for subsequent 4-fold extension by the *pksCT* gene, yielding an unreduced trimethylated pentaketide without the need of another PKS (21). The only structural difference between ascochitine and citrinin is that ascochitine has a sec-butyl group in the heterocyclic ring. As an alternative to the previously proposed biosynthetic scheme (9), the finding of the *orf7* in the ascochitine gene cluster in A. fabae suggested that the HR-PKS makes a diketide starter unit which is passed to the *pksAC* gene for further extension, producing ascochital and ascochitine via a mechanism analogous to that of asperfuranone biosynthesis (38). Notably, in the NR-PKS subclade that includes ascochitine (Fig. 1B), asperfuranone, azanigerone A, and bisvertinolone (a sorbicillinoid) are synthesized by the combination of an NR-PKS (AfoE, AzaA, and SorB, respectively) and a HR-PKS (AfoG, AzaB, and SorA, respectively) (31, 38, 39).

The ascochitine gene cluster also included a gene encoding a short peptide with a cupin domain (*orf2*) that is often found in secondary metabolite gene clusters (30, 40). The *orf11* gene encodes a fungus-specific Zn(II)2Cys6-type transcription factor and exhibited the least sequence similarity with the counterpart gene (*mrl3*) in the citrinin gene cluster. Unlike the *mrl3* gene, the deduced amino acid sequence of the *orf11* gene lacked a DNA-binding domain and contained only the conserved domain (termed “middle homology region”) that is commonly found in Zn(II)2Cys6-type transcription factors (41). Interestingly, the *sol4* transcription factor with only the middle homology region was shown to regulate the entire solanapyrone gene cluster in A. rabiei (29). This type of transcription factor without an apparent DNA-binding domain may activate genes by interacting with cognate repressor proteins, enabling timely induction of the repressed gene clusters.

When A. lentis and A. lentis var. *lathyri* are sexually crossed *in vitro*, they produce viable progeny, and segregation of molecular markers is normal, indicating that no intrinsic mating barrier exists between these taxa (42). However, pathogenic specialization of these two recently diverged species is evident (42). Although molecular phylogenies of protein-coding genes cannot clearly differentiate A. lentis from A. lentis var. *lathyri* (99.6% to 100% similar) (42), the two SNPs that caused a nonsense mutation in each taxon were consistent within each taxon, suggesting a prezygotic mating barrier and a lack of gene flow between the two probably sympatric species. Despite the presence of the nonsense mutation, transcripts of the *pksAC* gene in A. lentis isolate AL4 were detected on the basis of transcriptome sequencing (RNA-seq) data (see Fig. S1 in the supplemental material), suggesting that the gene is still active and likely undergoing pseudogenization. Compounds other than ascochitine were identified from cultures of A. lentis and A. lentis var. *lathyri* (15, 16, 43). Among the diverse mechanisms that produce genetic diversity in fungal secondary metabolism, nonsense mutation would be the fastest and the most effective, as the whole biosynthetic pathway of a compound can be disrupted by a single point mutation and indels in the responsible gene cluster (32, 44–48).

10.1128/mSphere.00622-19.2 FIG S1 Expression of the *pksAC* gene in Ascochyta lentis isolate AL4. A schematic diagram of the *pksAC* gene is shown. Yellow arrows indicate exons, and black blocks represent RNA-seq reads mapped on the *pksAC* gene. Download FIG S1, PDF file, 0.01 MB. Copyright © 2019 Kim et al. 2019 Kim et al. This content is distributed under the terms of the Creative Commons Attribution 4.0 International license.

Production of ascochitine by P. clematidina was evident during the infection process, and the amount of ascochitine production in axenic culture was positively correlated with the ability to cause the disease among P. clematidina isolates (4). In contrast, there was no such correlation between the aggressiveness of activity on faba bean cultivars and the amount of ascochitine production among A. fabae isolates (49). In this study, we demonstrated that ascochitine was not involved in the pathogenicity of A. fabae. Solanapyrone, another phytotoxic compound produced by A. rabiei, was also proven not to be a pathogenicity factor (29, 50). Therefore, caution should be taken in claiming the involvement of secondary metabolites in pathogenicity based on *in vitro* tests of phytotoxicity.

It is always a challenging task to determine the role of secondary metabolites in an interaction with hosts or in the environment (51). However, the role of secondary metabolites may be deduced from their biological activity and spatiotemporal production patterns during the life cycle of the producing fungi. As with solanapyrone, ascochitine was produced during sporulation, while another polyketide, pinolidoxin, produced by A. pinodes, was detected mainly in young, advanced hyphae (52). Together with the reported antifungal activities, these observations suggest that ascochitine may play a role in competition with other fungi during saprobic growth stages in nature (2, 52). Genes involved in secondary metabolite production are generally believed to confer a fitness advantage to the producing organism (51, 53). Otherwise, the highly plastic fungal genomes would have purged out costly genes encoding megasize proteins such as PKS by selective pressure over the course of evolution. To end on a speculative note, some lineages of legume-associated *Ascochyta* may have lost the ability to produce ascochitine, a compound to which some saprobic fungi are insensitive (2). Studying the precise role of ascochitine in fungal ecology will provide an opportunity for tracing the fate of secondary metabolite biosynthesis gene clusters in species divergence.

## MATERIALS AND METHODS

### KS domain phylogeny.

The predicted KS domains of 51 PKS genes with known polyketide products plus a putative ascochitine PKS gene (*pksAC*) were aligned using the MUSCLE program (v3.8.31) (54) (see Text S1 in the supplemental material). Subsequently, spurious sequences or poorly aligned regions from the 52 KS domains were automatically removed, using the trimAl program (v1.2) (55) with the following option argument: “-gappyout.” A maximum likelihood phylogeny based on a general empirical model (WAG) (56) was constructed using the Phangorn R package (v2.4.0) (57). Nodal support was evaluated by 1,000 bootstrap replications using nearest-neighbor interchange rearrangements.

### Gene replacement.

The *pksAC* gene in A. fabae isolate AF1 (WT) was deleted, using the split marker method (58). The DNA fragments used to construct the split marker were amplified using double-joint PCR (59). In the first round of PCR, a 756-bp upstream region of the *pksAC* coding sequence was amplified using primer pair L5 and L3, and a 947-bp downstream region of the *pksAC* coding sequence was amplified using primer pair R5 and R3. A 1,372-bp *hph* cassette was amplified from pDWJ5 plasmid (60) using primer pair HYG-F and HYG-R. The L3 and R5 primers carried 27-bp sequence tails that overlapped the 5′ and 3′ ends of the *hph* cassette, respectively. In the second round of PCR, the flanking DNA fragments were fused to the *hph* cassette through PCR by overlap extension. In the third round of PCR, the resulting PCR product was used as a template to generate the split-marker constructs that were amplified using primer pair N5 and HY-R and primer pair N3 and YG-F for the upstream and downstream flanking DNA fragments, respectively. Preparation of fungal protoplasts and transformation were conducted as previously described (61) with a minor modification. Volumes of 1 to 2 μg of each upstream and downstream split-marker DNA construct were used for genetic transformation. Putative transformants were subcultured on peptone-dextrose agar (PDA) containing hygromycin B (200 μg/ml). The gene replacement was confirmed by PCR with primer pair L5 and R3. Primers used in this study are listed in Table S1 in the supplemental material.

10.1128/mSphere.00622-19.3 TABLE S1 Primers used in this study. Download Table S1, DOCX file, 0.01 MB. Copyright © 2019 Kim et al. 2019 Kim et al. This content is distributed under the terms of the Creative Commons Attribution 4.0 International license.

### LC-MS analyses.

The WT and Δ*pksAC* mutant were grown on PDA for 14 days. Whole-agar chunks with fungal colonies were excised, soaked in 10 ml of ethyl acetate (EtOAc) in 50-ml Falcon tubes, and incubated at 4°C overnight. An aliquot of 200 μl of the extracts was transferred to a microcentrifuge tube, evaporated to dryness, reconstituted with 30 μl of MeOH, and subjected to LC-MS analyses. Chromatographic separation was achieved using an Acquity ultraperformance liquid chromatography (UPLC) system (Waters Corp., Milford, MA, USA), as previously described (50). MS analysis was performed on an inline Synapt G2-S high-definition mass spectrometry (HDMS; Waters Corp.) time of flight mass spectrometer using the positive-ion electrospray mode for data collection. The UV data were acquired using a wavelength of 210 to 400 nm, resolution of 1.2 nm, and 20 points/s with an Acquity photodiode array detector (Waters Corp.).

### Synonymous-nonsynonymous analysis.

SNAP (v2.1.1) (62) was used to estimate synonymous and nonsynonymous substitution rates and to calculate the ratio of synonymous to nonsynonymous substitutions between *pksAC* homologs in A. fabae AF247/15, A. viciae-villosae AV22, A. lentis AL4, and A. lentis var. *lathyri* ER1415 isolates. Since the genome assembly in the *pksAC* gene locus in A. lentis var. *lathyri* ER1415 was incomplete (with two N stretches [176 bp and 111 bp] of gap sequence in the coding region), the corresponding 96 codons from the other *pksAC* genes were excluded in this analysis.

### Pathogenicity tests.

Two faba bean strains (accession no. P1 667234 and PI 358266) were planted in Deepots (Stuewe & Sons, Inc., Corvallis, OR, USA) (6 by 25 cm) in a growth chamber. The inoculum was prepared by harvesting conidia (asexual spores) from 2-week-old fungal colonies on V8 agar media and was adjusted to 2 × 10^5^ conidia/ml. Two-week-old plants were sprayed with inoculum until runoff (approximate 2 ml per plant) and were immediately covered with an inverted translucent plastic cup to form a minidome to produce uniformly high levels of relative humidity for 24 h to facilitate infection. Plants were then placed in a growth chamber (Conviron model PGR 15, Winnipeg, MB, Canada) with settings of 12 h day (20°C) and 12 h night (16°C) at 100% relative humidity. Control plants were sprayed with water but were otherwise treated in the same manner as the inoculated plants. At 2 weeks after inoculation, disease scores were determined and numbers of lesions were measured, as previously described (63).

### MALDI imaging analysis.

Ascochyta fabae isolate AF1 and A. viciae-villosae isolate AV1 (33) was grown on half-strength PDA in which a microscopic slide was embedded. After 2 weeks of growth, the embedded slide with the fungal colony growing on the thin layer of PDA (about 2 mm in diameter) was removed from the plate and dried for 1 h at 65°C for MALDI imaging analysis. The dried specimen on the slide was sprayed with 20 mg/ml of 2,5-dihydroxybenzoic acid (in a mixture that also contained 0.1% trifluoroacetic acid and 50% methanol) as a matrix, using a TM-sprayer system (HTX Technologies, Carrboro, NC, USA) with the following parameters: 12 passes; track spacing, 1.2 mm; nozzle temperature, 70°C; linear velocity, 110 cm/min; flow rate, 60 μl/min. A selected area of the specimen was analyzed on a 9.4T Fourier transform ion cyclotron resonance (FTICR) MS instrument (Bruker Daltonics, Billerica, MA, USA) in positive-ion mode. Data were acquired using ftmsControl software (Bruker Daltonics). Approximately 100 shots per spot were acquired with a Smartbeam II Nd:YAG laser using repetition rate of 1 kHz. Image acquisition was carried out using flexImaging 4.1 (Bruker Daltonics), and data were normalized to the total ion current. Ascochitine standard compound purified in our previous study (8) was spotted, and reference MS signals were obtained in a simultaneous run.

### Data accessibility.

The genome assemblies for A. fabae isolate AF247/15 (GCA_004335285.1), A. lentis isolate AL4 (GCA_004011705.1), and A. viciae-villosae AV22 (deposited as ONG-16-641; GCA_004335205.1) were retrieved from the NCBI database. The ascochitine gene cluster *orf1* to *orf11* in A. fabae isolate AF247/15 was deposited in NCBI, and the data are accessible through accession no. MN052622 to MN052632.
