# Supplementary material for: Identification of a Polyketide Synthase Gene Responsible for Ascochitine Biosynthesis in Ascochyta fabae and Its Abrogation in Sister Taxa
Source: mSphere. 2019 Sep 25;4(5):e00622-19. doi: 10.1128/mSphere.00622-19 (PMC6763771; doi:10.1128/mSphere.00622-19)
Supplement: TEXT S1 [file mSphere.00622-19-s0001.docx]

**Text S1.** The deduced amino acid sequences of the KS domains of fungal PKS genes.

>ACTTS3_ACT-toxin

IAVIGSACRFPGGANSPHKLWELLRDPRDILREFPDDRLVLSKFYNGNANHHGSTNVRNRSYLLSEDIRAFDAPFFHINPREADGMDPAQRILLEAVYEALEAAGYTMEQMQGTHTSVFVGVMNSDWWDLQMRDTETIATHAATGTARSIVSNRISYVFDLKGVSMTIDTACSSSLVALHQAVQSLRSGESTAAIVGGANILLDPAMYIAESTLQMLSPESRSRMWDKSANGYARGEGCAAVFLKPLTRAIADGDHIECVIRETGVSSDGRTQGITMPSAAAQAALIKSTYRSAGLDPLADRCQYFECHGTGTPAGDPIEAQAIAEAFFSHSGEDAEIYVGSIKTVIGHLEGCAGLAGLLKASLAIQNRTIPANMLFNDLNPLIGPYYRNLKILQAAKPWPQDIHGPRRASVNSFGFGGTNAHVILES

>AfoE_asperfuranone

IAIVGMSVKTAGADDLDEFVAMLKTGQSQHIPITRDRLMHDMLFRENADADPKRKFYGCFFRDGDAFDHKFFKRSPRESAAMDPQSRIVLQAAYQAVEQSGYFVEDHNGYTPDGRDKMHVGVYLGSCGVDYEHNISCYDPNAFTATGALKSFITGRVSHHFGWTGPSCRNLLSGECTAALAGGSNTVTNMNWFQNLAAGSFVSPTGQCKPFDDDADGYCRAEGAAFVYLKRLSDALRDGNQVIATIAASAVYQNENCTPLFVPNSPSLSHLFKDVMRQAKVTANDVSLVEAHGTGTPVGDPAEYESILAALGGPSRKKKLPIGSVKGHIGHTEGASGAIALVKIIMMMREGFIPPQASFKTMNKKIPVKADDNIEVVTRLRAWEEERKTALLNNYGACGSNASMIVTQ

>AfoG_asperfuranone

AIIGLSCKFAGSADSPEKLWEMLAEGRNAWSEIPESRFNHKAVYHPDSEKLGTTLDPQFRFQLESVYEALENESTAGLTIPSIAGTNTSVYAGVFTHDYHEGLIRDEDKLPRFLPIGTLSAMSSNRISHFFDLKGASVTVDTGCSTALVALHQAVLGLRTREADMSIVSGCNIMLSPDMFKVFSSLGMLSPDGKSYAFDSRANGYGRGEGVATIIVKRLADALRDGDPVRGVIRESYLNQDGKTETITSPSQEAQEALIKECYRRAGLSPSDTQYFEAHGTGTPTGDPIEARSIASVFGKNREQPLRIGSVKTNIGHTEAASGLAGLIKVVLAMEKGFIPPSVNFEKPNPKLKLDEWRLKVADTLEKWPAPAERPWRASVNNFGYGGTNSHVIVE

>AptA_asperthecin

AVVGMACRMPGGGNDTELFWEILEQGRDVHTTVPADRFDLSTHYDPSGKTDNAATTPYGNFVDKPGLFDAGFFNMSPKEAEQTDPMQRLALVTAYEALEMAGVVPGRTASSNPKRIGTYYGQASDDWRELNASQNIGTYAVTGGVRAFGNGRINYYFKFPGPSFNVDTACSSGLAAVQVACSALWAGEADTVLAGGLNIITDPDNYAGLGCGHFLSKTGQCKVWDETADGYCRADGIGSVVIKRLEDAEADNDNIIAVVLSAATNHSAEAISITHPHAGNQKDNYRQVIDMAAVNPLDVSYIELHGTGTQAGDAVESESVLDVFAPRSPPRRPDQLLQLGAVKSNIGHGEAAAGIASFLKVLLMYQKNMIPAHIGIHTVINPTIPKDLEQRRVRLTQTNTPWPRLPGKKRIAMVNSFGAHGGNTTVLLED

>ACAS_atrochrysone

AIVGMSCRLPGGATNTEKFWDVLEQGLDVYRTIPPDRFDVNTHYDPAGKRVNASHTPYGCFIEEPGLFDAPFFNMSPREAQQTDPMQRLALVTAYEALERAGYVPNRTPATNKHRIGTFYGQASDDYREVNTAQDVDTYFITGGCRAFGPGRINYFFKFWGPSYSIDTACSSSLATVEAACTSLWNGSTDTAVVGGVNVLTNSDAFAGLSRGHFLSKIPGACKTWDCNADGYCRADGVISLVMKRLEDAQADNDNILGVILGAATNHSADAVSITHPHAGAQAHLFRDVLRNAGVDSHDVSYVELHGTGTQAGDFEEMKSVTDVFAPLTKRRSPNQPLYVGAVKANVGHGEAVAGVTALLKVLLMLQKSVIPPHVGIKNSINPQIPKDLDKRNLHIPYEKQSWKSTPGKSRIAVVNNFSAAGGNTSVVLEE

>AusA_austinol

IAVTGMACRYPQADSVEELWRILDLGQCTVSPMPNSRLKSGSLQREPKGPFFGNYLARPDAFDHRFFGISAREAESMDPQQRVLLQVAYEAMESAGYCGLRRSKLPDDIGCYVGVGCDDYSENVGSRNATAFSATGTLQAFNSGRISHYFGWSGPSVTVDTACSSAAVAIHLACQAIRTNDCAIAVAGGVNIMTDPRWSQNLAGASFLSPTGASKAFDADANGYCRGEGAGLLVLRPLEAALRDGDPIHAVITGTSVNQGANCSPITVPDSNSQRSLYLKALSLSGLTPDVVGYVEAHGTGTQVGDPIEFESIRKTFSGPNRATKLYVGSIKDNIGHTETSSGVAGMLKTILMIQKRRIPKQANFRRLNPRITLNERNHIEIPTQSIDWEAEKRVAMVTNYGAAGSNAAIVLRE

>AbPks9_depudecin

IAVVGMGCRLPGDVSSPSDFWRLMMEKRSGQTPKVPSSRFNIDAHFHPDNDRPGSFHVYGGYFINETLQEFDPAFFGITPVEATWMDPQQRKLLEVVYEAFESAGLTLDQLSGSDTACFMATFTADFQQMSFKEPSFRHSLAATGVDPGLLSNRVSHVFNLRGPSIVVNTACSSSVYALHNACNALRNHECSAAVVGGSNLILTVDQHMNTAKLGVLSPTSTCHTFNSYANGYGRAEGVGAIYLKRLSDAVKDGDPIRGVIRSSATNNNGRAPAVGITYPGFDGQRNVMMHAYQRSGLDPMLTGYFECHGTGTAIGDPLEVHAVSDVMNANRTEADGPLQMGAVKTNIGHSEAASGLSAVIKAILIAERNIIPPTRGLTDPNPKIDWKGWQINVPTESMTIPKHLPITRISVNSFGYGGTNAHTIIES

>OrsA_lecanoric_acid

IAVVGMSGRFPNSDTLDEFWRLLETATTTHQVIPESRFNVDDFYDPTRAKHNALLARYGCFLKNPGDFDHRLFNISPREAMQMDPVQRMLLMTTYEALEMAGYSPPTPAAPGDSEQAPPRIATYFGQTIDDWKSINDQQGIDTHYLPGVNRGFAPGRLSHFFQWAGGFYSIDTGCSSSATALCLARDALTAGKYDAAVVGGGTLLTAPEWFAGLSQGGFLSPTGACKTYSDSADGYCRGEGVGVVILKRLADAVRSKDNVIAVIAGASRNCNAGAGSITYPGEKAQGALYRRVMRQAAVRPEQVDVVEMHGTGTQAGDRVETHAVQSVFAPSNGNQREKPLIVGALKANIGHSEAAAGIISLMKAILILQHDKIPAQPNQPIKMNPYLEPLIGKQIQLANGQSWTRNGAEPRYIFVNNFDAAGGNVSMLLQD

>AoiG_O-methyldiaporthin

IAIIGFSGRFPEADGLNEFWELLQQGLDVHKPIPADRFDLEAHYDATLREKNTSRIKHGCWIRSPGSFDARFFQMSPREACQTDPAQRLALLTAYEAMEMAGFVPDRTPSSQRDRVGVYYGMTSDDWREVNSSQDIDTYFIPGGIRAFVPGRINYFFKFSGPSITVDTACSSSLAAIHTACNALLNSDCDTALAGGTNILTNPDNFAGLDRGHFLSSTGNCKTFDDDADGYCRADGVGTVILKRLQDAIADNDPIFGVIVGARTSHSAEAVSITRPLADAQAHLFRKLLAESGIHPHEISYIEMHGTGTQAGDAVEMKSVLDSFARDDSRAPDRPLHLGSVKANVGHGESASGVTALIKVLLMMQKNRIPPHCGIKGRINRHFPTDMEYRNVHIPFMETDWTRPQEGKRRSFINNFSAAGGNTAVLVED

>AdaA_TAN1612

AIVGMACRMPGGANDLDLFWELLAQGRDTHTTVPADRFDLETHYDPTGETENATRTPFGNFIDQPGLFDAGFFNMSPREAEQTDPMHRLALVTAYEALEMAGIVSGRTPSSNPKRIATFYGQASDDWRELNASQNIGTYAVPGGERAFANGRINYFFKFGGPSFNLDTACSSGLAAVQAACSALWAGEADTVLAGGLNIITDPDNYAGLGNGHFLSRTGQCKVWDQSADGYCRADGVGSVVIKRLEDAEADNDNILAVVLSAATNHSAEAISITHPHAGAQKENYTQVLHQAAVNPLDISYVELHGTGTQAGDAQEAESVLDIFAPRNHRRRADQPLHLGAVKSNIGHGEAAAGIASLLKVLLMYQKNEIPAHIGIPTVINPAIPTDLEQRKVYLPRTKTAWPRAAGQIRRAIVNSFGAHGGNTTLVLED

>Pyr2_pyripyropene

VAIIGTGCRFPGGASSPAKLWELLRNPREIARKIPANRFNIDAFYHPDGDHHGTTNVQESYFLDEDVRAFDAAFFNISPTEAAAMDPQQRLLLETVYESLDAAGLRMDALQGSMTGVFCGALRNDYSQIQTMDPQALPAYMVTGNSPSIMANRISYYFDWRGPSMTVDTGCSSSLLAVHLGVEALQNDDCSLAVAVGSNLILSPNAYIADSKTRMLSPTGRSRMWDSQADGYARGEGVASVVLKRLRDAIVDGDPIECVIRASGANSDGRTMGITMPNAQAQQALILQTYARAGLSPQERPTDRCQYFEAHGTGTQAGDPQEAAAIHASFFGPKSVADPLDRLFVGSIKTVVGHTEATAGLAGLIKASLSLQHGMIVPNLLMQQLNPKIEAFAAHLCVPTECVPWPAVPEGCPRRASVNSFGFGGANVHVVLES

>Trt4_terretonin

IAVTGMACRYPQADSMEELWKILEQGHCTVSPMPKNRFKLDELQREPKGPFWGNFLSRPDTFDHRFFKISAREAESMDPQQRLLLQVAYEAIESAGYCGLRASQLPQDVGCYVGVGTEDYSENVASRNATAFSATGTLQAFNSGRVSHYFGWTGPSVTIDTACSSAAVAIHLACQALQTNDCSMAVAGGVNVMTDPRWSQNLAAASFLSPTGASKAFDADANGYCRGEGAGLVVLRPLEAALRDGDPIHAVITGTSVNQGANCSPITVPDSNSQTTLYLKALSISGIKPDVVTYVEAHGTGTQVGDPIEFQSIRKTFAVPHRTERLYVGSIKDNIGHTETSSGVAGMLKTILMLQKRRIPKQANFTRLNPMITLQKEDQIFIPVESTDWKAEKRVAMVTNYGAAGSNAAIVLQE

>LovF_lovastatin

IAMVGMGCRFGGGATDPQKLWKLLEEGGSAWSKIPPSRFNVGGVYHPNGQRVGSMHVRGGHFLDEDPALFDASFFNMSTEVASCMDPQYRLILEVVYEALEAAGIPLEQVSGSKTGVFAGTMYHDYQGSFQRQPEALPRYFITGNAGTMLANRVSHFYDLRGPSVSIDTACSTTLTALHLAIQSLRAGESDMAIVAGANLLLNPDVFTTMSNLGFLSSDGISYSFDSRADGYGRGEGVAAIVLKTLPDAVRDGDPIRLIVRETAINQDGRTPAISTPSGEAQECLIQDCYQKAQLDPKQTSYVEAHGTGTRAGDPLELAVISAAFPGQQIQVGSVKANIGHTEAVSGLASLIKVALAVEKGVIPPNARFLQPSKKLLKDTHIQIPLCSQSWIPTDGVRRASINNFGFGGANAHAIVEQ

>LovB_lovastatin

IVVVGSGCRFPGDANTPSKLWELLQHPRDVQSRIPKERFDVDTFYHPDGKHHGRTNAPYAYVLQDDLGAFDAAFFNIQAGEAESMDPQHRLLLETVYEAVTNAGMRIQDLQGTSTAVYVGVMTHDYETVSTRDLESIPTYSATGVAVSVASNRISYFFDWHGPSMTIDTACSSSLVAVHLAVQQLRTGQSSMAIAAGANLILGPMTFVLESKLSMLSPSGRSRMWDAGADGYARGEAVCSVVLKTLSQALRDGDTIECVIRETGVNQDGRTTGITMPNHSAQEALIKATYAQAGLDITKAEDRCQFFEAHGTGTPAGDPQEAEAIATAFFGHEQVARSDGNERAPLFVGSAKTVVGHTEGTAGLAGLMKASFAVRHGVIPPNLLFDKISPRVAPFYKNLRIPTEATQWPALPPGQPRRASVNSFGFGGTNAHAIIEE

>FSL1_fusarielin

IAIIGTGCRFPGGSNTASKLWDLLKDPKDVSKEVPEDRFNLDRFYHKDSSHHGTANVRRSYLLDEDVRLFDTQFFGISPGEAQAMDPQHRVLLEVVYEAIESAGKTIHGLHNSDTAVYVGLMCTDYYVIQAADLNSVPTYNATGVANSNASSRVSYFFNWHGPSMTIDTACSSSLVAVHEAVQALRNGTSRMAVACGTNLILSPLPFISESNLSMLSPTGKSRMWDADADGYARGEGVAAVVLKPLSAAIEDNDVIECIIREVGVNQDGKTRGITMPSAQAQASLIRQTYAKAGLDPATPEGRCQFFEAHGTGTPAGDPQEAEALKTAFFPNETDSVTNGTNGLLSEADNLLVGSIKTVIGHTEGTAGLAGLIKACMALKHGAVPPNLLFNRLNPALEPFTKHLSIPTSLTPWPTLLTNVPRRASVNSFGFGGTNAHAILEA

>ZEA1_zearalenone

IAIVGMAGRGPGSDNVEEFWNVIMSKLDLCEEIPEDRFNLSEFYRSKHDSGCTTTTKFGCFMDKPGHFDNRFFHISPREALLMDPGHRQFLMTTYEALEMAGYSDGATRAVDPARIATFFGQCNDDWHDVSHHTLGCDAYTLQGVQRAFGAGRIAFQFKWEGPTYSLDSACASTASSIHLACTSLLAKETDMAVAGAANVVGYPHSWTSLSKSGVLSDTGNCKTFRDDADGYCRADFVGTVVLKRLEDAIAHNDNILAVVAASGRNHSGNSSSITTSDAKAQEKLYRKMMHNARVSPNDISYVEMHGTGTKVGDPAEMGALASLFSHRRTPKPVVVGGVKANVGHSESKQAAGVASLLKCIMMFQKNILPPQAGMPHALNPNFPPLSEINIEIPSEPSTFESPVSQPRRILLNNFDAAGGNACILLED

>ZEA2_zearalenone

VAIVGLACRFPGDATSPSKFWDLLKSGKDAYSETTDRYNAQAFYHPNSKRQNVLPVTGGHFLKQDPHVFDAAFFNITAAEAISLDPKQRIALEVAYEAFENAGKPLKQVAGTTTACFVGSSMSDYRDAVVRDFAHNPKYHVLGTCEEMIANRISHFFDIHGPSATVHTACSSSLVAIHLACQSLLSGDAEMALAGGVGMILTPDGTMQLNNLGFLNPEGHSRSFDKDAGGYGRGEGCGILVLKKLDKAIQDGDNIRAVIRASGVNSDGWTQGVTMPSSEAQAALIKHVYETRGLDYGATQYVEAHGTGTKAGDPVETGAIHRTIGQGASKNRKLWVGSVKPNIGHLEAAAGVASVIKGVLAMENSLIPPNIHFASPNPEIPLDEWNMAVPTKLTPWPAARTKRMSVSGFGMGGTNGHVVLEA

>AUR1_aurofusarin

IAIVAMSGRFPDAADLGEFWDLLYKGRDVHRQIPEDRFNAELHYDATGRRKNTSKVMNGCFIKEPGLFDARFFNMSPKEAEQSDPGQRMALETAYEALEMAGIVPDRTPSTQRDRVGVFYGMTSDDWREVNSGQNVDTYFIPGGNRAFTPGRLNYFFKFSGPSASVDTACSSSLAALHLACNSLWRNDCDTAIAGGTNVMTNPDNFAGLDRGHFLSRTGNCNTFDDGADGYCRADGVGTIILKRLEDAEADNDPILGVILGAYTNHSAEAVSITRPHAGAQEYIFSKLLRESGTDPYNVSYIEMHGTGTQAGDATEMTSVLKTFAPTSGFGGRLPHQNLHLGSVKANVGHGESASGIIALIKTLLMMEKNMIPPHCGIKTKINHHFPTDLTQRNVHIAKVPTSWTRSGQANPRIAFVNNFSAAGGNSAVLLQD

>BIK1_bikaverin

IAIIGFSGRFPEADNLDEFWDLLIRGLDVHKPVPEERFARDHYDPTGQRKNTSQVQYGCWLKSAGYFDTQFFHMSPKEAMQTDPAQRLALLTAYEALEMAGVVPDRTPSTQRNRVGVYYGTTSNDWGEVNSSQDVDTYYIPGANRAFIPGRVNYFFKFTGPSIAVDTACSSSLAAINLAITSLKNRDCDTAIAGGTNVMTNPDNFAGLDRGHFLSRTGNCKAFDDGADGYCRADGIGTLILKRLPDAIADSDPIFGVILGAHTNHSAESVSITRPLADAQEYLFKKLLNETGIHPHDVSYVEMHGTGTQAGDAVEMRSVLNSFAFDHSRPRDKSLYLGSVKANVGHAESASGVLAIIKVLLMMQKNTIPPHCGIKTKINQGFPKDLDHRGVRIALKDSVDWSRPEGGKRRVLVNNFSAAGGNTSLLLED

>FUM1_fumonisin

VAIVGMGMRLPGGIHTPDELWGMLVEKRSTRCEIPPTRFSVDGFHSPSSKPGSIAMRHGHFLDDKDDLHRLDTSFFSMGMTEVSDIDPQQRMLLEVAYECMQSSGQTNWRGSNIGCYVGVWGEDWLDLHSKDLYDSGTYRVSGGHDFAISNRISYEYDLKGPSFTIKAGCSSSLIALHEAVRAIRAGDCDGAIVAGTNLVFSPTMSVAMTEQGVLSPDASCKTFDANANGYARGEAINAIFLKPLNNALREGDPIRALVRATSSNSDGKTPGMSMPSSESHEALIRRAYGEVFLDPKDTCFVEAHGTGTSVGDPLEATAIARVFGGSSDNKLYIGSVKPNLGHSEGASGVSSVMKAVLALENRTIPPNINFSTPNPKIPFSEMNMAVPVDAIPWPRDRPLRVSVNSFGIGGANAHCIIE

>MlcB_compactin

IAIVGMGCRFAGDATSPQKLWEMVERGGSAWSKVPSSRFNVRGVYHPNGERVGSTHVKGGHFIDEDPALFDAAFFNMTTEVASCMDPQYRLMLEVVYESLESAGITIDGMAGSNTSVFGGVMYHDYQDSLNRDPETVPRYFITGNSGTMLSNRISHFYDLRGPSVTVDTACSTTLTALHLACQSLRTGESDTAIVIGANLLLNPDVFVTMSNLGFLSPDGISYSFDPRANGYGRGEGIAALVIKALPNALRDQDPIRAVIRETALNQDGKTPAITAPSDVAQKSLIQECYDKAGLDMSLTSYVEAHGTGTPTGDPLEISAISAAFKGHPLHLGSVKANIGHTEAASGLASIIKVALALEKGLIPPNARFLQKNSKLMLDQKNIKIPMSAQDWPVKDGTRRASVNNFGFGGSNAHVILES

>MlcA_compactin

IVVVGSGCRFPGGVNTPSKLWELLKEPRDVQTKIPKERFDVDTFYSPDGTHPGRTNAPFAYLLQEDLRGFDASFFNIQAGEAETIDPQQRLLLETVYEAVSNAGLRIQGLQGSSTAVYVGMMTHDYETIVTRELDSIPTYSATGVAVSVASNRVSYFFDWHGPSMTIDTACSSSLAAVHLAVQQLRTGESTMAVAAGANLILGPMTFVMESKLNMLSPNGRSRMWDAAADGYARGEGVCSIVLKTLSQALRDGDSIECVIRETGINQDGRTTGITMPNHSAQEALIRATYAKAGLDITNPQERCQFFEAHGTGTPAGDPQEAEAIATAFFGHKDGTIDSDGEKDELFVGSIKTVLGHTEGTAGIAGLMKASFAVRNGVIPPNLLFEKISPRVAPFYTHLKIATEATEWPIVAPGQPRRVSVNSFGFGGTNAHAIIEEY

>StcA_sterigmatocystin

AIVSMSGRFPEAPSTDSFWDLLYKGLDVCKEVPLRRWDVKTHVDPSGKARNKGATRWGCWLDFAGEFDPRFFSISPKEAPQMDPAQRMALMSTYEAMERGGIVPDTTPSTQRNRIGVFHGVTSNDWMETNTAQNIDTYFITGGNRGFIPGRINFCFEFSGPSYSNDTACSSSLAAIHLACNSLWRGDCDTAVAGGTNMIFTPDGHTGLDKGFFLSRTGNCKAFDDAADGYCRAEGVGTVFIKRLEDALAENDPILATILDIKTNHSAMSDSMTRPFKPAQIDNMSALLSTAGISPLDLSYIEMHGTGTQVGDAVEMESVLSLFAPDETFRPRDKPLYVGSAKANIGHGEGVSGVTSLIKVLLMMKNDTIPPHCGIKPGSRINRNYPDLPARNVHIAFEPKPWPRTDTPRRVLINNFSAAGGNTAVLVED

>DsPksA_dothistromin

AIVSMSGRFPEAQSTDAFWDLLYKGLDVVKEVPKRRWDVETHVDPTGRARNKGATKWGCWLDFAGEFDPRFFSISPKEAPQMDPAQRMALMSTWEAMERGGIVPDTTPSTQRNRIGVFHGVTSNDWMETNTAQNIDTYFITGGNRGFIPGRINFCFEFSGPSFTNDTACSSSLAAIHLACNSLWRGDCDTAVAGGTNMIFTPDGHAGLDKGFFLSRTGNCKPFDDKADGYCRAEGVGTVMVKRLEDALADGDPILGTILDAKTNHSAMSDSMTRPFVPAQIDNMEACLSTAGVDPTSLDYIEMHGTGTQVGDAVEMESVLSVFAPNEQFRGKDQPLYVGSAKANIGHGEGVSGVTSLIKVLLMMQNNTIPPHCGIKPGSKINHNYPDLAARNVHIAFEPKPFLRREGKLRRVLINNFSAAGGNTALLIED

>AflC_aflatoxin

AIVSMSGRFPESPTTESFWDLLYKGLDVCKEVPRRRWDINTHVDPSGKARNKGATKWGCWLDFSGDFDPRFFGISPKEAPQMDPAQRMALMSTYEAMERAGLVPDTTPSTQRDRIGVFHGVTSNDWMETNTAQNIDTYFITGGNRGFIPGRINFCFEFAGPSYTNDTACSSSLAAIHLACNSLWRGDCDTAVAGGTNMIYTPDGHTGLDKGFFLSRTGNCKPYDDKADGYCRAEGVGTVFIKRLEDALADNDPILGVILDAKTNHSAMSESMTRPHVGAQIDNMTAALNTTGLHPNDFSYIEMHGTGTQVGDAVEMESVLSVFAPSETARKADQPLFVGSAKANVGHGEGVSGVTSLIKVLMMMQHDTIPPHCGIKPGSKINRNFPDLGARNVHIAFEPKPWPRTHTPRRVLINNFSAAGGNTALIVED

>MpPksCT_citrinin

IAVIGMACRLPGAEDHEGFWEILKTGQSQHREVPEDRFGMATAWREADKRKWYGNFIDNYDTFDHKFFKKSPREMASTDPQHRLMLQVAYQAVEQSGYFRNNGTNRRIGCFMGVGNVDYEDNIACYPANAYSATGNLKSFLAGKISHHFGWTGPSLTLDTACSSSSVAIHQACRSILSGECNGALAGGVNVITSPNWYHNLAGASFLSPTGQCKPFDAKGDGYCRGEGVGAVFLKRLSSAIADGDQVFGVIASTKVYQNQNCTAITVPNAISLSELFTDVVRQARLEPKDITLVEAHGTGTAVGDPAEYDGIRAVFGGPIRSDVLSLGSVKGLVGHTECASGVVSLIKTLLMIQQGFIPPQASFSSINPSLNAKAEEKIEISTRLKPWDAPFRAALINNYGASGSNASMVVTQ

>PePksCT_citrinin

IAVIGMACQLPGAGDHEGFWEILKSGQSQHKEVPEDRFSLATAWREADKRQWYGNFIENYDTFDHKFFKKSPREMASTDPQQRLMLQVAYQTVEQSGYFREDGPNRRIGCFMGVGNVDYEDNIACYPANAYSATGNLKSFLAGKISHHFGWTGPSLTLDTACSSSSVAIHQACRSILSGECNGALAGGVNVITSPDWYHNLAGASFLSPTGQCKSFDAKGDGYCRGEGVGAVFLKRLSSAIADGDQVLGVIASTKVYQNQNCTAITVPNSGSLSELFTDVVRQARLEPRDISLVEAHGTGTPVGDPAEYDGIRAVFGGPIRSDILSLGSVKGLLGHSECASGVVSLIKILLVIQQGFIPPQGSFSKINPSLNAKDKDRIDISTRLKPWDAPFKAALINNYGASGSNASMVVTQ

>AfPksAC_ascochitine

IAVIGMACNVPGGEDMDEFWKILVAGKSQHEELPRGTGRFEFETPWREPYTKTKWYGNFIKDYDVFDHKFFKKGPREMLNTEPQHRLLLHAAYQTLEQSGYFSKPDYDKHIACFLGPGHVDYASSVNCYAPNAYTATGNLKSMCAGKISHHFGWTGPILTLDTACSSSCVAIHYACRSILSGEVSAALAGGSNVLSSVEWYENLSGAQFLSPTGQCKPFDAKADGYCRGDGIGLVFLKKLSTALADGDQVYGVIAGSKVYQNVGSTTITVPNADSLATLFKDITKQARIDPAKVSVVEAHGTGTPVGDPAEYEAICRIFGGSQRTDVLSLSSVKGLFGHTEGASGVCSLLKVLLMMHENAIPPQASFGSMNPGLKATTQDNIEVPTRLTPWKPNTQIALINNYGASGSNSSLVVTE

>Sol1_solanapyrone

IAIVGMGCRWPGGVRDASGLWELLKNKRSGYREFGDHRFSRKGFHHPNSEHPGTVATKGGFLLAEDPRLFDHAFFGIGSLEVETMDPSQRKLLEVVYEAFENSGEPWDSFSGSTTGVFVGNFSSDHLIIQGRDTDHPRPYASVGTGTSILSNRINYIFNLRGPSVTIDTACSSSMYALHLAISAIRNGDCDSAIVAASNTIIDPSTQLMMTKLGVLSPTSTSHTFDSSADGYARGEGFSALYLKRMSTAVDGDYPIRALVRGSALNANGRTGGITHPGREGQEAVIRKAYENAGNLPMKDTTFFECHGTGTPVGDPIEISAIGNVFGSATTPEKPLLVGSIKTNIGHTEPASAIAGIMKVVLALENGFIPPSIGIKKLNPNLDLKGGRINILTENTPWPDGRVRRASVNSFGYGGANGHCIIDD

>PhnA_herqueinone

IAIVGYSGRFPSAASNDAFWELLRSGQDVHREVPRDRFEWEKYYDPTGKKKNTSRVKYGCWIDEPGVFDTRFFNMSPKEAENTDPAQRLAITTTYEAMEMAGMVRNRTASTQQDRIGVFFGTTSDDWREVNSGQDVGTYFIPGGNRAFVPGRISYFFRFSGPSLSIDTACSSSFAAIQAACSYLWRGECDAAIAGGTNVLTSPDNFAGLDRAHFLSTTGNCNAFDDEASGYCRSDAVGSVVLKRLEDAEADNDPIFGVILGTNTNHCGQTESITRPHEGDQISVFKNIIRHSGIDPTDVSYIEMHGTGTQAGDATEMNSVLSAFVPKYKRTEMSPQRPLFIGSAKANIGHAESASGVSSLIKVMEMMKHNEIPPHCGIKNRINHNYPLDLAQRGVNIAFEVKPWLRENSNGGKRRVFLNNFSAAGGNTAMLIED

>FsPksN_red_pigment

IAIVGYSGRFPDSASNEAFWELLRAGKDVHREVPEDRFNWKTHYDPTGKTKNTSRVKYGCWIEEPGLFDARFFNISPREAENTDPAQRLAITTVYEAMEMAGMVPNRTPSTQQDRIGCFFGTTSDDWREVNSGQDVDTYFIPGGNRAFVPGRISYFFRFSGPSLSMDTACSSSFAAIQTACTYLWRGECDTAVAGGTNVLTNPDNFVGLDRGHFLSTTGNCNAFDDGASGYCRADAVGSVILKRLEDAEMDNDPIFGIISGATTNHCGQTDSITRPHEGDQTSVFKRIIRHAGVNPLDVSYVEMHGTGTQAGDATEMNSVLSVFVPGHQRMPRHPIYLGSAKANIGHAESASGVSSLIKVLMMMKHNEIPPHCGIKTKINHNYPLDLKDRNVNIASRPTPWLRDDAPCGKRLAFLNNFSAAGGNTAVLLED

>AbPksJ_alternariol

IAIVGFGFKFPQDITNAESLWKLLMERRSTMTEIPQNRWNIDGFYKEHGHRPGTVKNRGGHFLADDPARFDAPFFSVQPAEAECMDPQQRLLLETSYHALENAGIPMKAAMGTRTSVHVGCLLQEYSQISQRDSQMPGDYRIVGSSGLAMLANRLSWFYDFSGPSMTVDTACSGGLVAFHLACQELSAGSVDMSLVCGNNLCLLPDSTALLSSLNMMSKDSVCYSFDERASGYARGEGFGVLILKRLDTAIADGDTIRGVVRSTGCGQDGNTPSITSPSQSAQERLIRETYARAGLDLGDTRYFEAHGTGTPVGDPCEAAAISNVFSCRTPEDPIFVGALKSNMGHPEGASGIAGVIKTLLVLEKGIIPPNVYPERISPAVAAAGPNLKFPLVPATWPTDGIRRASVNSFGYGGTNAHVVLDD

>Hpm8_hypomycetin

VAIIGLACRFPGEATSPSKFWDLLKNGRDAYSPNTDRYNADAFYHPKASNRQNVLATKGGHFLKQDPYVFDAAFFNITAAEAISFDPKQRIAMEVVYEALENAGKTLPKVAGTQTACYIGSSMSDYRDAVVRDFGNSPKYHILGTCEEMISNRVSHFLDIHGPSATIHTACSSSLVATHLACQSLQSGESEMAIAGGVGMIITPDGNMHLNNLGFLNPEGHSRSFDENAGGYGRGEGCGILILKRLDRALEDGDSIRAVIRASGVNSDGWTQGVTMPSSQAQSALIKYVYESHGLDYGATQYVEAHGTGTKAGDPAEIGALHRTIGQGASKSRRLWIGSVKPNIGHLEAAAGVAGIIKGVLSMEHGMIPPNIYFSKPNPAIPLDEWNMAVPTKLTPWPASQTGRRMSVSGFGMGGTNGHVVLEA

>Hpm3_hypothemycin

IAIVGMAGRGPGCENVDEFWDVIMAKQDRCEEIPKDRFDINEFYCTEHGEGCTTTTKYGCFMNKPGNFDSRFFHVSPREALLMDPGHRQFMMSTYEALETAGYSDGQTRDVDPNRIAAFYGQSNDDWHMVSHYTLGCDAYTLQGAQRAFGAGRIAFHFKWEGPTYSLDSACASTSSAIHLACVSLLSKDVDMAVVGAANVVGYPHSWTSLSKSGVLSDTGNCKTYCDDADGYCRADFVGSVVLKRLEDAVEQNDNILAVVAGSGRNHSGNSSSITTSDAGAQERLFHKIMHSARVSPDEISYVEMHGTGTQIGDPAEMSAVTNVFRKRKANNPLTVGGIKANVGHAEASAGMASLLKCIQMFQKDIMPPQARMPHTLNPKYPSLSELNIHIPSEPKEFKAIGERPRRILLNNFDAAGGNASLILED

>GsfA_griseofulvin

IAVVGMSCRFPGSDTTEEFWERLMLGEDMHRHIPPDRFDVETHVDPTGKRHNTSKTSYGCFVDNPGLFDAMFFGMSPREAEQTDPMQRLALVTAYEALEKAGYVDGRGVIHRKRVGTFYGQASDDYREVNSGQEVGTYFIPGGCRAFGPGRINYFLNFWGPSFSVDTACSSSLAAIQAACSSLWSGDIDMAITGGMNILSNSDVYAGLSQGHFLSPTGGCKTWDEGADGYCRSDGVGSVVLKRLEDAEADNDNILAVVLSAATSHSAEAVSITHPHDAAQALLYNQIVRRAGIDPLEVGYVEMHGTGTQAGDPTEMRSVTSVFAPPHIQGSRPIPLHVGSVKANMGHGEAAAGIMAFVKTMLVFQNGIIPPHIGVKTGLNPALPDLDKAGVVIPFRAANWRPTGTKKRLAMVNNFGAAGGNTAMIIEE

>MapC_mycophenolic_acid

IAITGMACRYPNADTLAQLWDLLELGRCTVKSPPESRFHMSDLQREPKGPFWGHFLERPDVFDHRFFNISAREAESMDPQQRVALQVAYEAMESAGYLGWQPNGLSRDIGCYVGVGSEDYTENVASRNANAFSITGTLQSFIAGRISHHFGWSGPSISLDTACSSAAVAIHLACKALQTNDCKIALAGGVNVLTNPRVYQNLSAASFLSPSGACKPFDASADGYCRGEGAGLFVLRPLQDAIDNGDPILGVIAGSAVNQGSNNSPITVPDAEAQRSLYNKAMSLAGVSPDEVTYVEAHGTGTQVGDPIELDSLRRTFGGPQRRNSLHIGSIKGNIGHTETSSGAAGLLKTILMLQQQRIPRQANFNQLNPKVKSLTPDRLVIASESTEWASTERVAMVSNYGASGSNAALIVKE

>TropA_tropolone

IAIVGASIKVAGADDLEEFWEILSKGISQHKEVPPERFTFDTVYRDRDPKTKWYGNFLNDPDKFDHKFFKKSPREAESMDPQQRLLLQIAYQALEKGGYFHNAGPDQRIGCYMGVCAVDYENNLACYAPNAFTATSHLRGFIAGKVSHYFGWTGPALTIDTACSSSAVAVHLACQAILKGECTAALAGGTQILTSPLWFQNLAGASFLSKTGQCKPFDSKADGYCRGEAVGAVFLKKMSAALADGDQILGVISGTAVQQNENCTPIVVPNKPSLSDMFQSVIEKARLQPDHITVVEAHGTGTAVGDPVEYASVRDTLGGSKRTKKLFLGSAKGLVGHCESASGIISLVKVLLMIQKGMIPPQASFNTLNPATKATPADGIEISRQLTEWNAPFRAALINNYGASGSNASMVITQ

>VrtA_viridicatumtoxin

AIVGMACRMPGGANNVEEFWQLLEQGRDACTTVPPDRFDLETHYDPTGKTENAAQTPYGNFIDRPGYFDAAFFAMSPKEAEQTDPMQRLAIVTAYEAMEMAGLVIGRTQSTRRDRIGSYYGQASDDWRELNASQNIGTYAVPGGVRGFTVGRINYFFKLSGPCLCIDTACSSSMAAVHAACTALWAGDVDVALAGGVNIITDPDNYAGLGNAHFLSPTGQCKVWDKGADGYCRAEGIGSVVIKRLEDAEADNDNILAVVLSAATNHCADAISITHPHAGHQKDNCRRVLRKAGVSPMQVSYVEMHGTGTQAGDAIESESVLDVFAPLKPLRRPDQRLHLGAVKSNIGHGEAAAGISSLIKMLLMFQKNAIPPHIGIRTEMNPQLPKDLGRRNAGLVFETTPWLRPEGKKRISVVNSFGAHGGNTTLLLED

>Rdc1_radicicol

IAIVGMSGRGPRSDNIDEFWDVIMQKQDTCTEVPKDRFDIDEFYCEEHGKGNKICTMTTKYGCFMDKPGHFDARFFHISPRESMLMDPGHRHFLMSSYEALEMAGYSDGPTKLTDPNRIAAFYGQVTDDWHDQSHPTLGCDAYTLQGVQRAFSSGRLAWQFKWEGPTYSLDSACASTTAAIHLACMSLLSNDIDMAVAGASNILNYPHSFACLSKSGVLSDTGNCKPYRDDADGYCRADFVGSVVLKRLEDAVADNDNILAVIASSGRNHSGNSTSITSSDPGAQERLFRKVLRNANVSPDDISYVEMHGTGTPVGDPAEMSAVGNVFKHRRRADGPLPVGAVKANFGHSEGAAGMASLLKCIMMFKTDTIPPQAGMPHALNPNFPPLSELNVEIPAEPKEFKKTRSGEPRRILLNNFDAAGGNACLLLED

>Rdc5_radicicol

IAIIGMSCRFPGDAEDPLKFWDLLKEGREAYSEKTHRYNEEAFYHPGGQFNNKRQNVLPVKGGYMLKQDPYVFDAAFFNITAAEAISFDPKQRIAMEVTYEAFENAGMTLQKAAGTRTACYIGTSMSDYRDSIVRDFGNYPKYHLLGTSDEMISNRISHFFDLRGPSATIETACSSSHVATHIACQSIQSGESDMAVVGGIGMLLVPESTMQLNNLGFLSAFGQSRAFDASGAGYGRGEGCGIFILKRLDKAMEDGDTIRAIIRGSGVNSDGWTQGVTMPSGDAQASLIEYVYKSNGLDYEGTQYVEAHGTGTKVGDPTEAEALHRTIGQPTPKRKKLWMGSVKTNIGHLEAAAGAASMVKGVLAMEHGFIPPTLHFKNPNPAIKFDEWQLGVPTKLMPWPACQTRRMSTSAFGMGGTNAHLVLE

>TmPKS11_mitorubrin

IAVVGMSLKVAGADDTDEFWDLLCAGQSQHREVPRNRIKFDNDWREVGPKRKYFGNFLNDHDIFDQKFFKKSAREAASTDPQQRILLHVAYQALEQAGYFNSPEQDKRIGCFIGECANDYADNVACHQPNAFTATGNLKSFIAGKVSHYFGWTGTGLTLDTACSSSLVAVHLACKAILSGECNAALAGGVNMMNSALWFQNLAAASFLSPTGQCKPFDANADGYCRGEAVGVVFLKSMSAAIANGDQIIGTISSTGVSQNQNCTPIFVPNAPSLSTLFQDVIQDAQVDPKKISVVEAHGTGTQVGDPAEYDSIRRVLGGANLRSKPLAFGSVKGLVGHTEASSGLVSLIKILLMIQNKTIPPQASHESLNPHLNATADDKMEIITKKTTWDEDYRAALINNYGASGSNASAVVTE

>TmPKS12_mitorubrin

IAIVGAGCRLPGANSIDELWEILSAGSSRVEKLRSSRFDLSTVSRGSVGPDAKQTAKRELYGNFLDDVESFDSNFFGISPREAMYMDPQQRLLLETAYEALDGSGYLRTHRRGDFDNVGCFIGASYTEYLENTSSYNPTAYTATGTIRAFQSGRISYHFGWSGPSEVIDTACSASLVAVNRACKAIQSGECPMALAGGVNIITGVNNYFDLGKAGFLSTTGQCKPFDETADGYCRADGVGLVALKSLRQAVADGNNVMGVIMGVGTNQGGLSPAITVPYYRAQISLFKNVLNQSGLKSGQISYVEAHGTGTQVGDPIEISSVREVFGGSDRSEFVNMGSLKANVGHSETAAGIGSLMKVLAMLKHGKIPPLAGFKSLNPKIPALEPDYLRIPTELQDWNSSFRAACVNSYGAAGSNSALICG

>EncA_endocrocin

IAIVGMACRFPGGANDLNQFWDLLEQGADVHRRVPADRYDVESHTDTSGKSRNTSLTPFGCFIDQPGLFDAGFFDMSPREAMQTDPMHRLALMTAYEALEQAGFVPNRTESTHLKRIGTFYGQSCDDYREANAGQEVDTYYIPGGCRAFAPGRINYFFKFSGPSFDCDTACSSSLATIQMACTSLQHGDTNMAVAGGLNILTNSDGFAGLSRGHFLSKTGGCKTFDCNADGYCRADGIGSIVLKRLDDAQRDNDHIFGIILAAATNHSARAISITHPHAPSQAELYRDILTRAGVSPLDVDFIEMHGTGTQAGDSTEMESITSVFSPGVPKRSRPLYIGSVKANVGHGEAAAGVMSLIKVLLVLQRQAIPKHVGIKTALNPRFPNLDRLNVRIPHDQVPWPRSPTRKRYALVNNFSAAGGNTSLLIEE

>NscA_neosartoricin

LAIVGMACRLPGGANDPELFWELLEQGRDTLTTVPPDRFDLNTHYDPTGKTENATQTPFGNFIDRPGYFDAGFFNMSPREAEQTDPMHRLALVTAYEAMEMAGLVPGRTPSTRPNRIGTFYGQASDDWRELNASQNISTYAVPGGERAFANGRINYFFKFSGPSYNIDTACSSGLAAVQAACSALWAGEADTVIAGGLNIITDPDNYAGLGNGHFLSKTGQCKVWDKDADGYCRADGIGSVVIKRLEDAEADNDNILAVVLGARTNHSAEAVSITHPHAGAQKANYRQVLHQAGVNPLDVSYVELHGTGTQAGDAVESESVSDVFAPSMPRRRPDQRLYLGAVKSNIGHGEAAAGIASLLKALLVYQKNMIPKHIGIKTEINPIIPKDLDRRHVGLAMSNTPWPRPAGKKRLAVVNSFGAHGGNTTVLLED

>MdpG_emodin

IAIIGMSCRMPGGATDTEKFWELLEQGLDVARKIPADRFDVETHYDPKGKRVNTSHTPYGCFIDEPGLFDAPFFNMSPREAQQTDPMQRLAIVTAYEALERAGYVANRTPATNLHRIGTFYGQASDDYREVNTAQEISTYFIPGGCRAFGPGRINYFFKFSGPSFSCDTACSSSLATIQAACTSLWNGDTDMVVAGGMNVLTNSDAFAGLSHGHFLSKTPGACKTWDVNADGYCRADGIGSIVMKRLEDAEADNDNIIGIIRAAATNHSAEAISITHPHAGAQAYLYRQVMSSAGIDPLDVSFVEMHGTGTQAGDSVEITSITDIFAPITKRRSAQQPLHIGAVKANVGHGEAVAGVTALLKVLLMYQKNAIPPHVGIKNSLNPLFPKDLDKRNLHIPYQKVPWPRVKGKKRYAVVNNFSAAGGNTTVCLEE

>ChPks1_T-toxin

IAVVGMSFRGPGDATNVEKLLNMISEGRESRAEVQAKKWDPEGFYHPDSSRHGTHNVEYGHWFQQDVYNFDAPFFNVSPAEAAALDPQQRMLLECSYEAFENSGTPMSKIVGTDTSVFVSSFATDYTDMLWRDPESVPMYQCTNSGFSRSNLANRISYSFDLKGPSVLVDTACSGGLTALHLACQSLLVGDVRQALAAGSSLILGPEMMVTMSMMKFLSPDGRCYAFDERANGYARGEGVAVLLLKRLEDALADNDTIRAVIRGTGCNQDGKTPGITMPNSVSQEALIRSVYKKAALDPLDTTYVECHGTGTQAGDTTEASALSKVFSPGRRLPLLIGSVKTNIGHLEGASGLAGVVKSILMLEQGVILPNRNFERPNTKIPLEKWNLRVPTTLECWNNVKTRRVSINSFGYGGANVHAILES

>ChPks2_T-toxin

AIIGMSCRFPGKVASLEDFWDMLSNSKHGYRQFPRERFNWEAFYHPNQSRKDCIDVNCGYFLDGDIAEFDAQFFKMNGTDAASFDPQGRMILECVYEALENAGVPKESIVGSKVGVFSTSNTSDYTLSLKDDIYSMPALVGVLGHACMLSNIVSNTFDLKGPSVSIDTACSSAFYALQLASQSLRSGETEMCIVSGCALNISPWRWTMLSNLTMLNPDGLSKSFDPQADAGYVRGEGAASIIVKPLDAAIRDNDRVHCVLSDIGVNHNGRTNGYTLPDARMQASLMRELQVRLDIKPDEFGFVEAHAPGTRVGDPIEISALQEVFSTSARTLEDPLLIGSVKANVGHLESSSGFPSLIKAAMMLKKGLVVPNANFENESMNSHLKEKNMRVPISTQPWPKGKTYIAINNYGFGGSNSHCIVR

>DzmPks1_PM-toxin

IAIVGLSFRGPGDATSAENLLRMVAESRESRSPIPSQKWNASGHYHPDPSRQGSHIVEYGHWFQQDVYEFDAPFFNLSAVESAALDPQQRMLLECTYEAFENSGMPLNKLVGTDTSVFTAVFCTDYTDMLWRDPEMVPMYQCTNSGATRANMANRVSYSFDLKGPSITVDTACSGGLTALHLACQSLVTGESTQAVVSGSSLILGPETMVTMSMMRFLSPDGRCYAFDDRANGYARGEGVTVLLLKRLDDALANGDTIRAVIRGTGRNPDGKTTGIAMPSGLAQEALIRSVYAKTGLDLLDTAYIECHGTGTQAGDTTEARAISNVFGPGRQVPLAIGSVKTNIGHLEAASGLAGVLKCILMLENEIILPNRNFKHANLNIPLEEWKLRVPTTVEPWNSMTTRRASVNSFGYGGTNVHAILES

>AzaB_azanigerones

AIIGMACRFSGGATSPEKLWDMIVQRRSGWSEIPTSRFNANGLYHPNGERVGTTHVKGGHFLEDDIACFDAAFFGMASETASAMDPQYRMELEVVYEALESAGIPMESIKGTNTSVYGGVMFRDYHDTHSRDLDTLPRYFMTGNAATMASNRISHFYDLRGPSMTVDTGCSTSLTALHLACQNLRSGESNMSIVTGASLMINPDVFLSMSNIGFLSPDGISYAFDSRANGYGRGEGVGALLVKRLDDALRDGDSIRAIIRETGVNQNGKTPSITAPQQAAQEALIRQCYERVNLDPAQTTYVEAHGTGTPAGDPLEVGALAAALGGSRSAEHPLYLGSIKANIGHTEAASGVASIIKVALALEKGQIPPNTQLNTPNSELRLNDRNMEVPVSTQRWPVGKGPRRASVNNFGFGGSNAHAILES

>AzaA_azanigerones

IAVIGMSCKVAGADDVDEFWDLLCKAESQHQEVPKERFGFESAFREVDPTRKWYGNFINEHDCFDHKFFKKSAREIAATDPQQRQMLQVAYQAVEQSGYFTTPKSDKDRKIGCYIGVCAADYEYNVACHPPNAFMATGNLKSFVAGKISHWFGWTGPGLCIDTACSSSLVAVHQACQAILTGDCTAALAGGANIITHPLWYQNLAAASFLSPTGQCKPFDASADGYCRGEGFAAVFLKKMSAAIADGDMIIGSIKATAVNQNQNCTPVFVPNAPTLSDLFRDVLDRSQLTANQITVVEAHGTGTQVGDPAEYESIRNVLGGPSRSTPLLFGSVKGLVGHTECTSGAVSLVKTLLMQQHEAIPPQPSFDRLNPEIPVSESDNMQIATRFSPWTAEYRAALINNYGACGSNASMVVAQ

>SorB_sorbicillinoid

IAVIGMSCQVAGAQDLEQYWNILLEGRSQHKNLVPNERFAMETVFRPGQDGEDRKWYGNFIDDYDAFDYKFFRKSPREVLHMDPQQRLILQTAYQAVAQSGYYHRPGADRRIGCYIGCVANDYENNISHTSPTAFSATGALRSYIAGKVSHYFGWTGPGMMLDTACSASTVAIDLACRAILSGDCSAALAGGTNFYSTPMFFQNLAAGSFLSPTGQCKPFDAKADGYCRGEAIGAVFLKKLSNAIADGDQILGVISATAINQNQNDTPIFVPNPSSLTNVFQNVVGKAGLEVNDISVVEAHGTGTPVGDPAEYDSIRQVFGGSVRAGLKPLQLGSVKGLIGHTEGASGVVALIKMLLMMQESRIPPQASFTSMSASIKASPADNMEITKAALPWEDESKVALINNYGAAGSNASMVIKQ

>SorA_sorbicillinoid

IAIIGMSAKFAGDATNTDNLWRMLIEGRSGWSPFPDSRFRSEGVYHPNNERLNSTHVKGAHFLAEDVGLFDAAFFGYSGETAASMDPQYRLQLESVYEALENAGLPLTKIAGSNTSVFTGVFVHDYRDGFLRDADNLPRLMATGTGVPMMANRVSHFFDLRGASMTIETACSSGMVAVHQAVQSLRTGEADMSIVGGANLTLNPDMFKALGSAGFLSADGKSYAFDSRASGYGRGEGVGTLVVKRLSDALAAGDPIRAVIRESMLNQDGKTETITSPSLEAQEALVRGCYQKAGLDPRETQYFEAHGTGTQAGDTIEAQGIATVFASRQEPLLIGSIKTNVGHTEAASGLASIIKTALAMENGVIPPSINFEKPNPKISLDDWNLKLVREVETWPAGPIRRASINNFGYGGSNAHIILED
