# Supplementary figures and images for: Identification of a Polyketide Synthase Gene Responsible for Ascochitine Biosynthesis in Ascochyta fabae and Its Abrogation in Sister Taxa
Source: mSphere. 2019 Sep 25;4(5):e00622-19. doi: 10.1128/mSphere.00622-19 (PMC6763771; doi:10.1128/mSphere.00622-19)

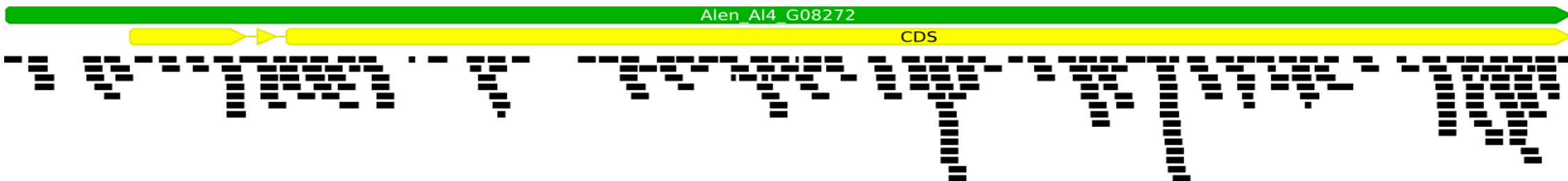

Supplement: FIG S1 [file mSphere.00622-19-sf001.pdf]
