# Supplementary material for: Identification of a Polyketide Synthase Gene Responsible for Ascochitine Biosynthesis in Ascochyta fabae and Its Abrogation in Sister Taxa
Source: mSphere. 2019 Sep 25;4(5):e00622-19. doi: 10.1128/mSphere.00622-19 (PMC6763771; doi:10.1128/mSphere.00622-19)
Supplement: TABLE S1 [file mSphere.00622-19-st001.docx]

**Table S1.** Primers used in this study

| **Primers** | **Sequences (5’🡪 3’)** |
| --- | --- |
| L5 | CACGGGGCTGAGTTCCATATATC |
| L3 | TTGACCTCCACTAGCTCCAGCCAAGCCTGCCTTGATTCCTTCCGGGGTGT |
| R5 | GCAAAGGAATAGAGTAGATGCCGACCGGGCAAAGCCTGTGTTTTCAAGGG |
| R3 | CGTTGCTTACCCCATCGAATTCC |
| N5 | CTGACCATCCACCTTCGATC |
| N3 | ACGCAGGTGTAGCAGCTCTTC |
| HYG-F | GGCTTGGCTGGAGCTAGTGGAG |
| HYG-R | CGGTCGGCATCTACTCTATTCCTT |
| YG-F | CGATGTAGGAGGGCGTGGATATGTCC |
| HY-R | GTATTGACCGATTCCTTGCGGTCCGAA |
| SNP1 | CTTTGCAGTGTCTACATCGTCGAC |
| SNP2 | CCACTGACAAGTCCTTTGATCTGC |
